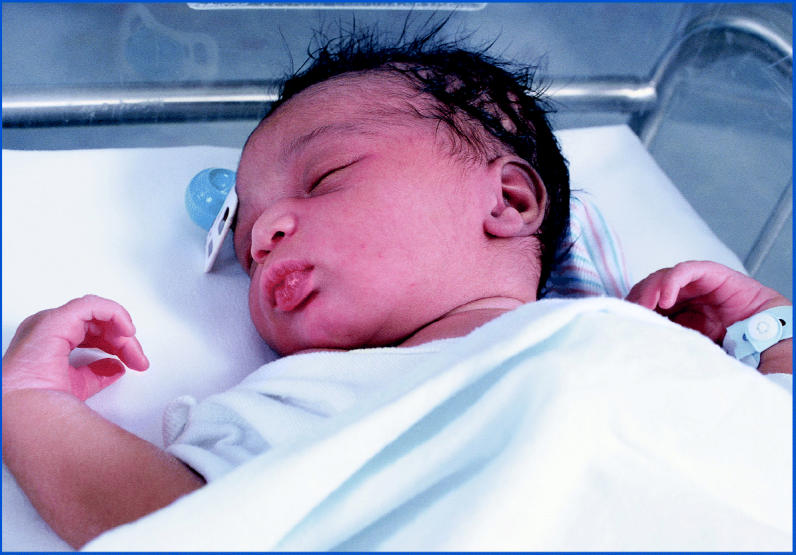# Headliners: Chromosomal Damage: Prenatal PAH Exposure Causes Genetic Changes in Newborns

**DOI:** 10.1289/ehp.113-a237

**Published:** 2005-04

**Authors:** Jerry Phelps

Bocskay KA, Tang D, Orjuela MA, Liu X, Warburton DP, Perera FP. 2005. Chromosomal aberrations in cord blood are associated with prenatal exposure to carcinogenic polycyclic aromatic hydrocarbons. Cancer Epidemiol Biomarkers Prev 14:506–511.

Research has suggested that *in utero* exposure to pollutants can cause DNA damage, chromosomal changes, and increased risk of childhood cancers such as leukemia. Other studies have indicated that pediatric leukemia is initiated prior to birth. Now NIEHS grantee Frederica Perera and colleagues at Columbia University demonstrate for the first time that prenatal exposure to polycyclic aromatic hydrocarbons (PAHs) causes chromosomal changes that have been linked to leukemia and other cancers.

PAHs are a group of more than 100 different chemicals that are formed during the incomplete burning of almost any organic substance, including coal, oil, and gasoline. PAHs can cross the placenta, and many have been shown to cause cancer in animal studies.

The Columbia researchers used a technique known as fluorescence *in situ* hybridization to identify and count the number and types of chromosomal changes that occurred in a subset of 60 African-American and Dominican newborns from the Columbia Center for Children’s Environmental Health Prospective Cohort Study in New York City. They measured chromosomal changes in cultured white blood cells from umbilical cord blood. The researchers assessed prenatal exposure via questionnaires administered to the babies’ mothers during the third trimester of pregnancy, and by analyzing data from personal air monitors that the mothers wore during their third trimester.

The researchers found that exposure to airborne PAHs was significantly associated with stable chromosomal aberrations. They also observed that African-American babies had a higher frequency of chromosomal changes than Dominican babies, suggesting the presence of either other unmeasured factors or variations in susceptibility.

These findings suggest that prenatal exposures may cause the cytogenetic damage that has been related to increased cancer risk, the researchers write. This information has global importance because air pollution conditions similar to those in New York City exist in other cities in the United States and the world. Although further research is necessary to confirm these findings and to estimate the increase in cancer risk from the exposures, these results demonstrate once again the importance of protecting children from avoidable harmful exposures.

## Figures and Tables

**Figure:**